# Development and Structural Characterization of UTE‐156, a Covalent Inhibitor of the VCP/p97 AAA+ ATPase

**DOI:** 10.1002/advs.202520545

**Published:** 2026-03-07

**Authors:** Daniela Tamayo‐Jaramillo, Subramanya Hegde, Xuan Jia, Kimberly Coffman, Hariprasad Vankayalapati, David Bearss, Kevin B. Jones, Alex W. Stark, Peter S. Shen

**Affiliations:** ^1^ Department of Biochemistry University of Utah Salt Lake City Utah USA; ^2^ University of Utah Therapeutics Accelerator Hub University of Utah Salt Lake City Utah USA; ^3^ Huntsman Cancer Institute University of Utah Salt Lake City Utah USA; ^4^ Department of Orthopaedics University of Utah Salt Lake City Utah USA

**Keywords:** chemical probes, covalent, cryo‐EM, inhibitors, UTE‐156, valosin‐containing protein, p97, AAA ATPase

## Abstract

The AAA+ ATPase valosin‐containing protein (VCP/p97) is a central regulator of protein homeostasis that is well characterized for its role in extracting and remodeling ubiquitinated substrates. Dysregulation of VCP activity contributes to the pathogenesis of neurodegenerative diseases and cancer, making it an important therapeutic target. Here, we report the development and characterization of UTE‐156, a novel covalent small‐molecule inhibitor that modifies Cys522 within the D2 ATPase domain of VCP. UTE‐156 potently inhibits VCP ATPase activity, while losing activity against a C522A mutant, supporting a covalent mechanism of action. High‐resolution cryo‐electron microscopy (cryo‐EM) structures reveal that UTE‐156 occupies the D2 nucleotide‐binding site, sterically blocking ATP binding and inducing conformational remodeling of the pocket. Biochemical and cell‐based assays demonstrate strong inhibitory potency but limited solubility and rapid metabolic turnover. These pharmacochemical limitations preclude immediate therapeutic use but underscore its value as a chemical probe. Together, these findings establish UTE‐156 as a powerful tool for dissecting VCP function and provide a framework for future optimization of covalent modulators of protein homeostasis.

## Graphical abstract

The AAA+ ATPase Valosin‐containing protein (VCP/p97) regulates protein homeostasis by unfolding ubiquitinated substrates. Here, we describe UTE‐156, a novel irreversible covalent inhibitor that modifies Cys522 in the D2 ATPase motor domain. Although its pharmacochemical limitations preclude immediate therapeutic use, UTE‐156 serves as a valuable chemical probe and framework for developing covalent modulators of VCP function.

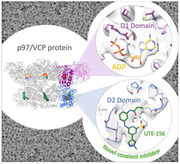

## 1 Introduction

Maintaining protein homeostasis is essential for cellular health and survival and requires the timely recognition and removal of misfolded or damaged proteins from various cellular compartments [1]. The VCP (valosin‐containing protein) enzyme is a central regulator of these processes in eukaryotes [2]. VCP (also known as p97) belongs to the superfamily of **A**TPases **a**ssociated with diverse cellular **a**ctivities (AAA+) and functions as a homo‐hexameric double‐ringed ATPase that extracts and unfolds ubiquitinated substrates from protein complexes, membranes, or ribosomes to facilitate their degradation or other remodeling events essential for proteostasis. This unfoldase activity underlies its indispensable role across multiple protein quality control pathways, including the clearance of stalled translation products from ribosomes through ribosome‐associated quality control (RQC) [3, 4, 5, 6], segregation of misfolded proteins through the endoplasmic reticulum associated degradation (ERAD) pathway [2], turnover of chromatin‐bound [7] and mitochondrial substrates [8], regulation of endosomal [9] and Golgi trafficking [10], and initiation of autophagic responses [11, 12].

VCP protomers contain an N‐terminal domain (NTD) that mediates adaptor binding and substrate recruitment, followed by two conserved AAA+ ATPase domains, D1 and D2 [1, 13]. These domains are stacked to form the hexameric core, with D1 contributing to substrate insertion into its central pore and D2 serving as the main motor for ATP‐dependent substrate unfolding. This modular architecture enables VCP to couple substrate engagement with mechanical translocation through its central axial pore [1, 13].

Given its broad role in maintaining cellular proteostasis, it is not surprising that the dysregulation of VCP is linked to a range of human diseases. Over 100 missense mutations in VCP have been identified in autosomal dominant multisystem proteinopathies, which manifest as degenerative conditions such as frontotemporal dementia, inclusion body myopathy, Paget's disease of bone, amyotrophic lateral sclerosis (ALS), and Charcot‐Marie‐Tooth disease [14]. These mutations are thought to impair coordinated ATP hydrolysis or disrupt adaptor interactions, leading to defective substrate processing.

Tumor cells are highly dependent on maintaining imbalanced proteostasis networks, and inhibition of VCP's ATPase activity has emerged as an attractive strategy to exploit this vulnerability [13]. The therapeutic relevance of targeting proteostasis has been clinically validated by the success of proteasome inhibitors, such as bortezomib [15], which have significantly improved outcomes in multiple myeloma and mantle cell lymphoma. Carfilzomib, a next‐generation irreversible covalent proteasome inhibitor, has further advanced this therapeutic class by providing durable responses to treat relapsed or refractory multiple myeloma, highlighting the promise of covalent inhibition in proteasome‐targeted cancer therapies [16]. These agents disrupt protein degradation and induce proteotoxic stress by promoting the accumulation of misfolded proteins, leading to cell death. Given VCP's upstream role in substrate extraction and proteasomal delivery, its inhibition offers a complementary and potentially synergistic approach to proteostasis disruption in cancer therapy [13].

**FIGURE 1:**
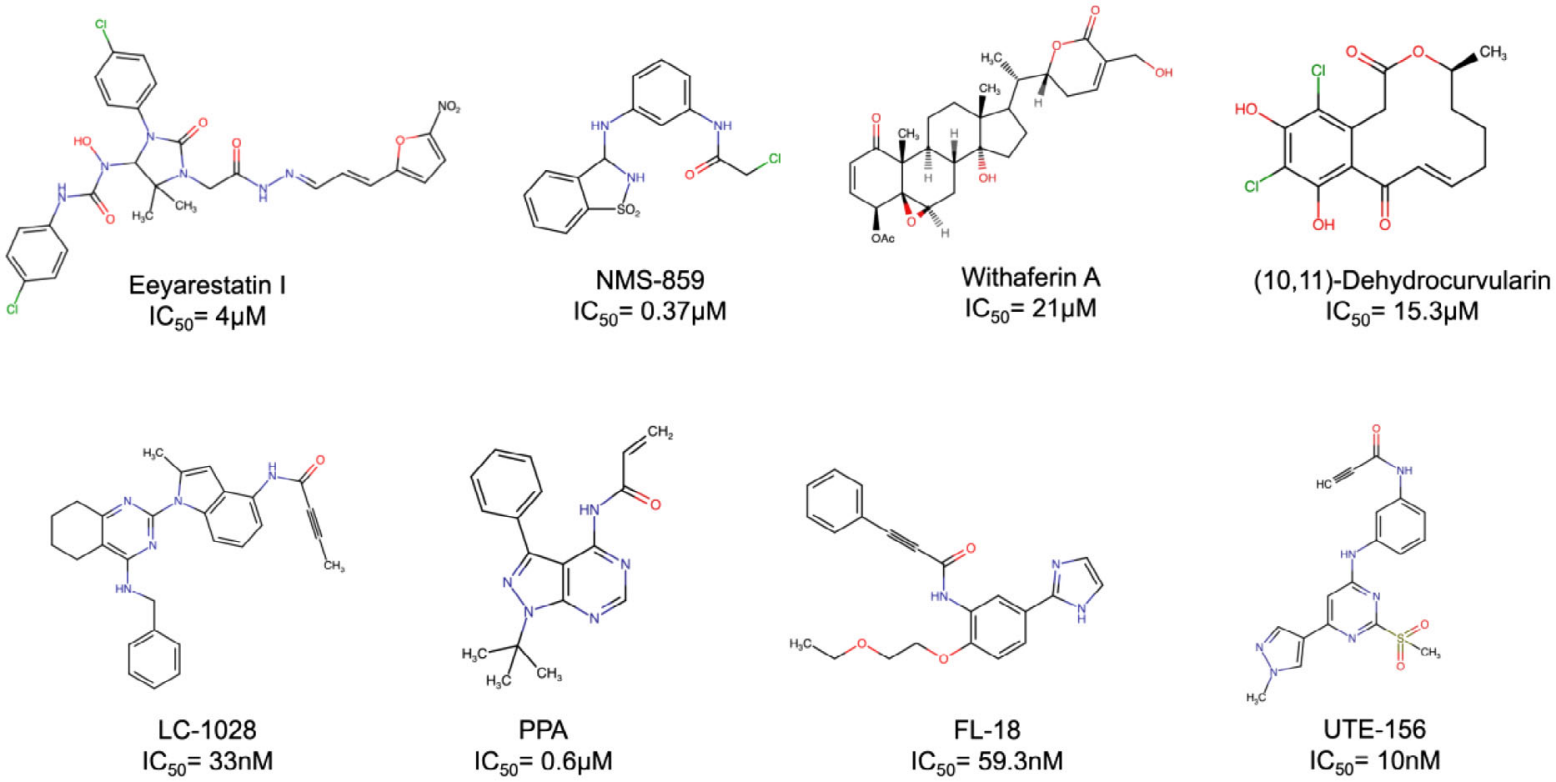
Covalent VCP inhibitors reported in the literature, arranged chronologically from left to right: Eeyarestatin I [25, 26], NMS‐859 [17], Withaferin A [31], (10‐11)‐Dehydrocurvularin [32], LC‐1028 [33], PPA [30], FL‐18 [34], and UTE‐156 (this study) with corresponding IC_50_ values.

Several classes of VCP inhibitors have been developed, including allosteric, competitive, and covalent compounds [13]. Allosteric inhibitors, such as NMS‐873 [17] and UPCDC30245 [18, 19, 20], stabilize VCP in an inactive conformation, whereas competitive inhibitors such as CB‐5083 [21, 22, 23, 24] bind the nucleotide‐binding site and block ATP hydrolysis. Covalent inhibitors, including Eeyarestatin I [25, 26] and NMS‐859 [17], irreversibly modify reactive residues within or near the ATPase domains (Figure 1). Despite their potent biochemical activity, none of these compounds have advanced to clinical use.

CB‐5083, the most clinically advanced candidate, entered Phase I trials but was withdrawn due to dose‐limiting ocular toxicity attributed to off‐target inhibition of phosphodiesterase 6 (PDE6), a photoreceptor‐specific enzyme essential for vision [27]. Subsequent optimization of the CB‐5083 scaffold led to the development of CB‐5339, a second‐generation derivative that advanced to clinical evaluation and demonstrated improved selectivity and in vivo activity [28, 29]. Nonetheless, challenges including off‐target effects, limited metabolic stability, and the emergence of resistance mutations in VCP continue to motivate the exploration of alternative inhibitory mechanisms.

These setbacks underscore the need for potent, selective, and mechanistically diverse VCP inhibitors. Expanding the repertoire of small molecules that engage VCP through distinct binding modes could not only improve therapeutic prospects but also provide valuable chemical tools to dissect VCP's complex biology across cellular contexts.

More recently, the development of PPA (*N*‐(1‐(*tert*‐butyl)‐3‐phenyl‐1*H*‐pyrazolo [3,4‐*d*]pyrimidin‐4‐yl)acrylamide) was reported as a covalent inhibitor that modifies Cys522 within the D2 ATPase domain of VCP [30]. This work demonstrated that Cys522 is a viable site for covalent modification and that engagement at this position can impair VCP function in cells. In parallel, LC‐1028, a structurally distinct electrophile‐containing inhibitor, was reported to exhibit high biochemical potency and has been proposed to engage Cys522. However, for both compounds, mechanistic conclusions were inferred primarily from biochemical and modeling analyses, without direct structural visualization of covalent engagement. As a result, the atomic basis of Cys522‐directed covalent inhibition has remained unresolved.

Here, we report the discovery and characterization of UTE‐156, a novel covalent small‐molecule inhibitor that selectively targets Cys522 in the D2 ATPase domain of VCP (Figure 1). We demonstrate that UTE‐156 covalently modifies this residue, resulting in potent inhibition of ATPase activity. Our cryogenic electron microscopy (cryo‐EM) work provides the first high‐resolution structures of VCP covalently modified by an ATP‐binding inhibitor. These structures reveal how UTE‐156 sterically blocks ATP binding and induces conformational remodeling of the D2 nucleotide pocket. Although limited solubility and metabolic stability preclude immediate therapeutic application, UTE‐156 establishes a valuable chemical probe for dissecting VCP function and provides a framework for the rational design of next‐generation covalent inhibitors targeting protein homeostasis.

## 2 Results

### 2.1 Development of a Potent VCP Inhibitor

Guided by the goal of developing irreversible covalent VCP inhibitors, we used a VCP crystal structure (PDB ID: 6MCK) [23] to perform structure‐ and fragment‐based virtual screening of approximately 500,000 commercially available small‐molecule fragments. Fragment hits were filtered using Rule‐of‐Three criteria and selected to resemble elements of known pyrimidine‐based VCP inhibitors, while incorporating altered chemical connectivity to explore new binding modes. This strategy yielded multiple potent scaffolds positioned in close proximity to Cys522. Cys522 is a residue located within the Walker A motif of the D2 ATPase domain. Although this residue is not essential for ATP hydrolysis, its position within the nucleotide‐binding pocket makes it an attractive site for selective covalent inhibition of VCP.

Because covalent engagement of Cys522 was a central design objective, a range of electrophilic warheads was evaluated. While several electrophiles, including acrylamides and other substituted electrophiles, produced weak or predominantly reversible inhibition, compounds bearing an alkynyl ketone warhead exhibited robust potency and irreversible covalent behavior. This warhead was therefore selected for further optimization, ultimately leading to the development of UTE‐156.

**SCHEME 1:**
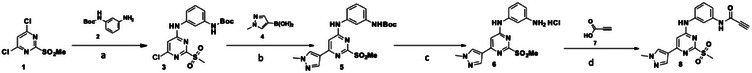
Synthesis of UTE‐156 (Compound 8). Reagents and conditions: (a) 2,6 lutidine, DMF, 0°C to rt, 30 min; (b) PdCl_2_(dppf), K_2_CO_3_, Dioxane, H_2_O, 60°C, 2 h; (c) HCl (4.0 m), Dioxane, rt, 3 h. (d) propioplic acid, EDCI, pyridine, DMF, 0°C‐ rt, 1 h.

The chemical synthesis of UTE‐156 (compound **8**) is summarized in Scheme 1. The synthesis began with 4,6‐dichloro‐2‐(methylsulfonyl) pyrimidine (compound **1**), which was coupled to tert‐butyl (3‐aminophenyl)carbamate (compound **2**) in the presence of 2,6 lutidine and DMF to yield intermediate **3**. This was followed by a Suzuki cross‐coupling with 1‐methyl‐1H‐pyrazol‐4‐yl boronic acid (compound **4**) to generate intermediate **5**. Boc deprotection of intermediate **5** with HCl produced compound **6,** which was then coupled to propiolic acid (compound **7**) using EDCI to yield the final product, compound **8** (UTE‐156). The identity of compound **8** (UTE‐156) was confirmed by ^1^H and ^13^C NMR, High‐resolution mass spectrometry (HRMS), and Differential scanning calorimetry (DSC) (Figures S1, S2 and S3).

### 2.2 UTE‐156 Binds Covalently to Cys522

To confirm the covalent modification of VCP by UTE‐156, we performed intact mass spectrometry on purified human VCP. Incubation with UTE‐156 resulted in a single adduct corresponding to a mass shift of +395 Da, consistent with the mass of one UTE‐156 molecule, thus indicating complete modification of the protein (Figure S4).

We next carried out peptide mass fingerprinting (PMF) analysis using LC‐MS/MS to map the site of modification. Among 11 cysteine‐containing peptides detected, only Cys522 displayed high‐efficiency covalent modification (98.2%), while all other cysteines showed negligible modification (<1%) (Table 1). These data demonstrate selective covalent engagement of Cys522 within VCP; however, we note that this selectivity reflects target‐site specificity rather than a comprehensive assessment of proteome‐wide cysteine reactivity.

**TABLE 1:** Mass of cysteine containing peptides with and without covalent modification. Yellow rows indicate detected peptides modified by UTE‐156. Blue row indicates Cys522‐containing peptide modified by UTE‐156.

**Sequence**	**Site of modification**	**Sequence (start‐end)**	**Observed m/z**	**Modification**
EAVCIVLSDDTCSDEKIR	C77	66–83	703.9981	
LGDVISIQPCPDVK	C105	96–109	770.9064	
LGDVISIQPCPDVK	C105 + UTE‐156	96–109	940.4439	0.3%
VVETDPSPYCIVAPDTVIHCEGEPIKR	C174	165–191	771.1309	
EDEEESLNEVGYDDIGGCRK	C209	192–211	771.9964	
LADDVDLEQVANETHGHVGADLAALCSEAALQAIR	C415	390–424	918.9533	
LADDVDLEQVANETHGHVGADLAALCSEAALQAIR	C415 + UTE‐156	390–424	1003.7156	0.2%
GVLFYGPPGCGK	C522	513–524	626.3113	
GVLFYGPPGCGK	C522 + UTE‐156	513–524	795.8531	98.2%
AIANECQANFISIK	C535	530–543	789.9015	
AIANECQANFISIK	C535 + UTE‐156	530–543	959.4408	0.8%
QAAPCVLFFDELDSIAK	C572	568–584	962.48	
QAAPCVLFFDELDSIAK	C572 + UTE‐156	568–584	1132.0191	0.4%
MTNGFSGADLTEICQR	C691	678–693	900.4065	

### 2.3 UTE‐156 Inhibits the ATPase Activity of VCP

**FIGURE 2:**
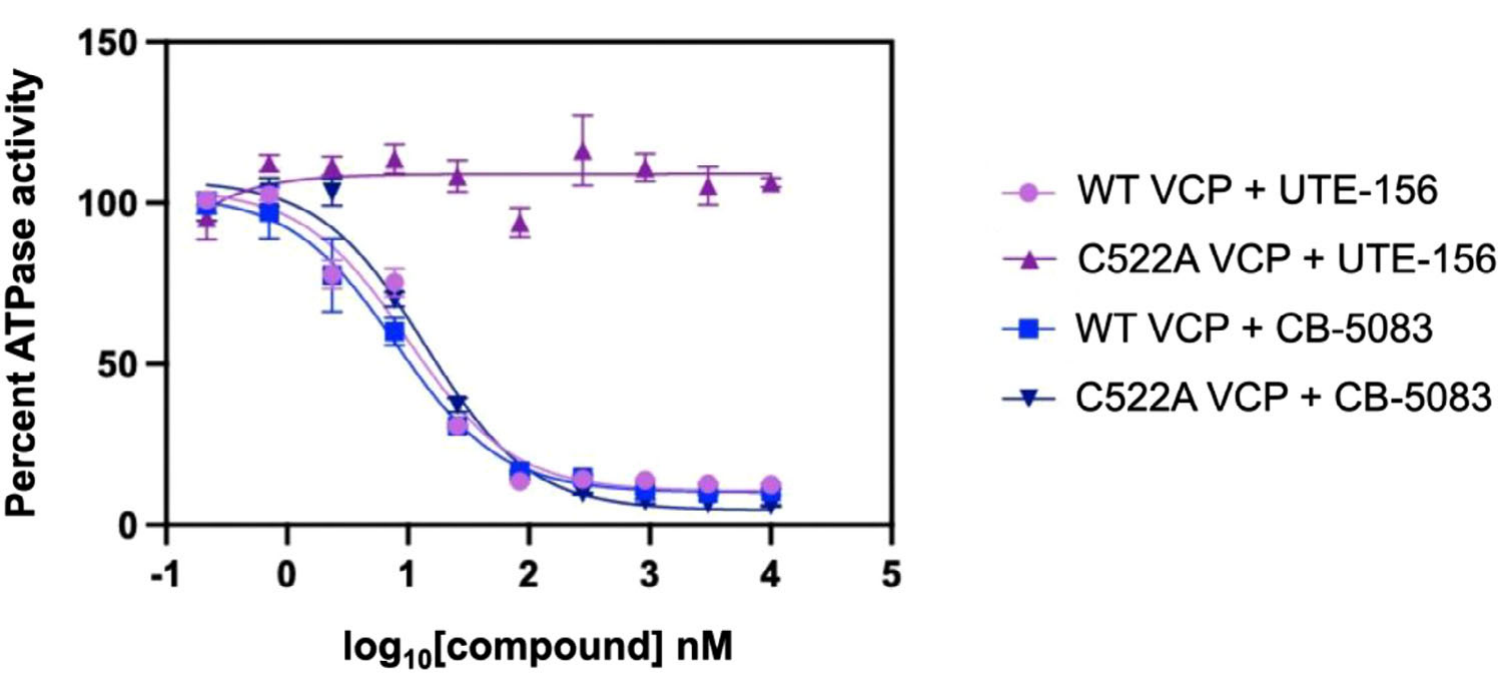
Dose titration in the ADP‐glo ATPase assay for VCP.

We next evaluated the effect of UTE‐156 on VCP enzymatic activity using an ADP‐Glo ATPase assay (Figure 2). VCP was preincubated with UTE‐156 for 60 min at room temperature before ATP addition, allowing time‐dependent covalent engagement. UTE‐156 potently inhibited wild‐type (WT) VCP with an IC_50_ of 10 nm, indicating strong target engagement. In contrast, the C522A mutant displayed markedly reduced sensitivity (IC_50_ > 10 µm), confirming that Cys522 is critical for compound activity.

To further distinguish scaffold‐derived activity from electrophile‐dependent inhibition, we evaluated UTE‐330 (Figure S5), an analog that retains the same core scaffold as UTE‐156 but lacks the alkynyl warhead. UTE‐330 exhibited minimal inhibition of VCP ATPase activity (Figure S5B), demonstrating that the potency of UTE‐156 arises primarily from covalent modification of Cys522 by the alkyne warhead.

For comparison, LC‐1028, a reported high‐potency VCP inhibitor containing an electrophilic moiety [33], was evaluated side‐by‐side with UTE‐156 under identical ATPase assay conditions. Under these conditions, LC‐1028 inhibited the ATPase activity of WT VCP without inducing a Cys522‐dependent loss of activity (Figure S6). Consistent with this observation, intact mass and PMF (MS/MS) analyses revealed no significant covalent modification, supporting a predominantly reversible mode of inhibition (Figure S6 and Table S2).

To assess whether UTE‐156 broadly inhibits AAA+ ATPases, we evaluated its effect on the structurally related AAA+ ATPase Spastin, which does not contain cysteine in its Walker A motif. Under identical assay conditions, UTE‐156 did not measurably inhibit Spastin ATPase activity, supporting target selectivity within VCP (Figure S7).

When evaluated under identical assay conditions, UTE‐156 inhibited VCP with apparent biochemical potency comparable to that of the reversible inhibitor CB‐5083 (Figure 2). However, because IC_50_ values alone do not capture the kinetics of irreversible covalent inhibition, mechanistic conclusions in this study are based on time dependence and direct evidence of covalent target engagement rather than numerical potency comparisons. These results establish UTE‐156 as a well‐characterized covalent inhibitor of VCP and provide a framework for expanding the chemical space of Cys522‐directed modulation.

Taken together, UTE‐156 exhibits robust biochemical inhibition of VCP and displays clear hallmarks of irreversible covalent engagement, including through its pronounced time‐dependent inhibition, loss of activity against the C522A mutant, and direct peptide‐level mass spectrometric evidence of stable covalent modification.

### 2.4 Cellular Activity and ADME Profile of UTE‐156

We next assessed the activity of UTE‐156 in cell‐based assays and profiled its physicochemical properties (Table 2). In A549 lung carcinoma cells, UTE‐156 reduced viability with an IC_50_ of 1.6 µm, indicating moderate antiproliferative activity. In contrast, HCT116 colorectal carcinoma cells showed minimal sensitivity (IC_50_ > 100 µm). This disparity underscores the distinction between biochemical potency and cellular efficacy and likely reflects compound‐specific properties such as solubility, cellular uptake, or intracellular stability.

**TABLE 2:** In vitro ADME results for UTE‐156.

Assay	Result for UTE‐156
ADP‐Glo IC_50_	10 nm
ADP‐Glo IC_50_ [C522A]	>10 𝜇m
CTG A549	1.61 𝜇m
CTG HCT116	100 𝜇m
logD	2.5
Solubility	0.83 𝜇m
LM Clearance m	36.9 𝜇l/min/mg
Heps Clearance mouse/human	935.35/110.6 𝜇l/min/10^6^cells
GSH t_1/2_	5.58 min
RBC binding (B/P)	0.77

To evaluate drug‐like properties, we characterized the physicochemical and ADME (Absorption, Distribution, Metabolism, and Excretion) properties of UTE‐156. The compound displayed a moderate LogD (pH 7.4) of 2.5, indicating balanced lipophilicity. However, UTE‐156 showed low kinetic solubility (0.83 µm), which may limit oral bioavailability and complicate formulation strategies. Additionally, high hepatic intrinsic clearance was observed in both mouse (935.35 µL/min/10^6^ cells) and human (110.6 µL/min/10^6^ cells) hepatocytes, suggesting rapid metabolic turnover and a likelihood of poor systemic exposure in vivo.

UTE‐156 also exhibited rapid glutathione reactivity (t_1/2_ = 5.6 min) and minimal red blood cell binding (blood‐to‐plasma ratio = 0.77). This rapid reactivity likely limits effective cellular exposure and may contribute to reduced antiproliferative activity observed in cell‐based assays, consistent with a trade‐off commonly encountered for electrophilic covalent inhibitors.

As observed for other VCP inhibitors, a substantial shift between biochemical ATPase inhibition and cellular potency was evident. This disparity likely reflects a combination of physicochemical and intracellular pharmacokinetic limitations rather than assay artifacts. No visible precipitation was observed under the cellular assay conditions used, suggesting that factors beyond gross insolubility contribute to the reduced cellular activity.

Together, these results demonstrate that while UTE‐156 is a potent covalent inhibitor of VCP in vitro and active in a relevant cancer cell line, its poor solubility and high metabolic clearance limit its current suitability for in vivo studies. These properties reinforce its value as a chemical probe rather than a therapeutic lead and highlight areas for future optimization.

### 2.5 Covalent Modification of Cys522 with UTE‐156 Impedes the Binding of ATP in D2

**FIGURE 3:**
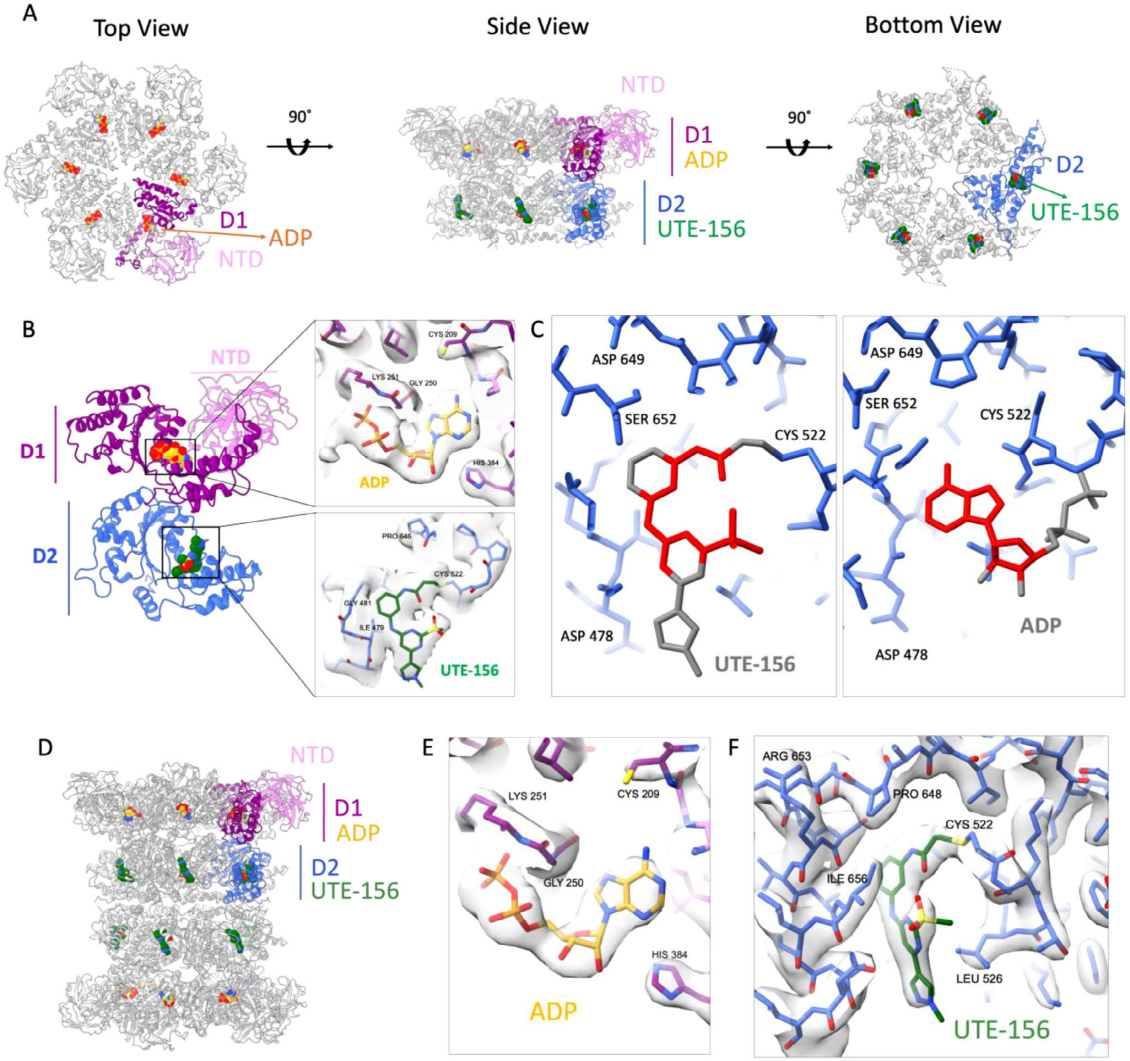
Structural characterization of UTE‐156 binding to VCP. (A) Model of VCP hexamer displayed from side, top, and bottom views. (B) Model of VCP protomer displayed from side view showing D1 and D2 motor domains and respective binding sites for ADP and UTE‐156. Insets correspond to (top) a close view of the D1 binding pocket with refined model for VCP (blue) and ADP (yellow) with cryo‐EM reconstruction density of VCP hexamer + UTE‐156 (gray mesh); and (bottom) a close view of the D2 binding pocket with refined model for VCP (blue) and UTE‐156 (green) with cryo‐EM reconstruction density of VCP hexamer + UTE‐156 (gray mesh). (C) (left) Model for UTE‐156 binding at the D2 domain pocket. (right) Model for canonical ADP binding at the D2 domain pocket (PDB ID: 5FTK) [18]. The covalent modification of Cys522 with UTE‐156 creates a steric hindrance that prevents the binding of ATP. Atoms that participate in clashes are shown in red, atoms that do not participate in clashes are displayed in gray. (D) Model of VCP dodecamer displayed from side view depicting an overview of the D1 and D2 motor domains highlighting ligand engagement. (E) Cryo‐EM reconstruction density of VCP dodecamer + UTE‐156 (gray mesh) showing a view of the D1 binding pocket with refined model for VCP (blue) and ADP (yellow). (F) Cryo‐EM reconstruction density of VCP dodecamer + UTE‐156 (gray mesh) showing a view of the D2 binding pocket with refined model for VCP (blue) and UTE‐156 (green). All maps shown in this figure are displayed at a contour level of 6 σ.

To elucidate the mechanism of VCP inhibition by UTE‐156, we determined the structure of the ligand‐bound complex using single particle cryo‐EM. Purified human WT VCP was incubated with UTE‐156, and the cryo‐EM reconstruction at 2.8 Å resolution revealed that the compound‐bound VCP retained its canonical homo‐hexameric architecture, composed of six protomers arranged in a double‐ring configuration (Figure 3A).

In the hexameric assembly, ADP was bound in the D1 domains, whereas clear density corresponding to UTE‐156 was present at Cys522, a residue located within the Walker A motif of the D2 ATPase domain (Figure 3B; Video S1), consistent with intact and peptide‐level mass spectrometry results. In contrast, analysis of a UTE‐156‐free (DMSO control) cryo‐EM map revealed ADP bound in both the D1 and D2 nucleotide‐binding pockets (Figure S8). These observations indicate that UTE‐156 binding is associated with the displacement of nucleotide from the D2 domain while preserving the native nucleotide state in D1.

**FIGURE 4:**
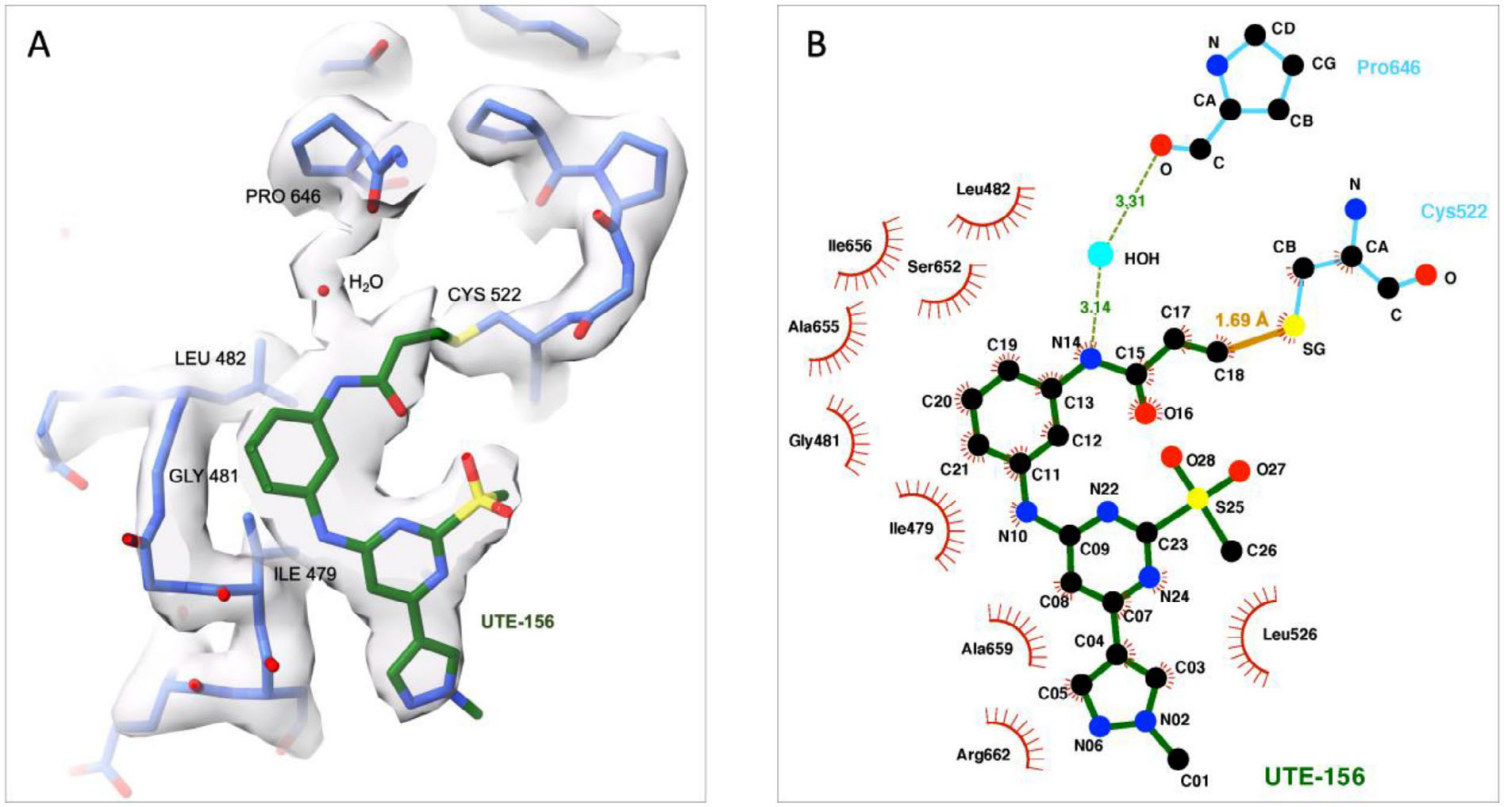
Stabilizing interactions of UTE‐156 within the D2 nucleotide‐binding pocket. (A) Cryo‐EM reconstruction density of VCP dodecamer + UTE‐156 (gray mesh) showing a view of the D2 binding pocket with refined model for VCP (blue) and UTE‐156 (green). The density map corresponds to the post‐processed, locally filtered reconstruction and is contoured at 6σ. (B) LigPlot+ [37]. representation of UTE‐156 in the binding pocket. The continuous maroon line represents the covalent bond formed between UTE‐156 (C18) and Cys522. Dashed green lines indicate the water mediated hydrogen bonds between UTE‐156 (N14) and Pro646. Dashed red lines denote van der Waals interactions between UTE‐156 and surrounding residues.

In the UTE‐156‐bound structure, the electrophilic alkyne carbon (C18) is positioned for nucleophilic attack by the thiol group of Cys522 (Figure 4). Surrounding residues within the nucleotide‐binding pocket undergo local rearrangements to accommodate covalent bond formation and ligand occupancy.

The covalent attachment of UTE‐156 to Cys522 completely occupies the nucleotide‐binding pocket. UTE‐156 occupies the region that would otherwise accommodate the adenine base and ribose of ATP, effectively blocking ATP access through steric hindrance (Figure 3C). Additionally, UTE‐156 engages the binding site in an orientation that is orthogonal to that of ATP, representing a distinct mode of interaction that allows UTE‐156 to reach further into the core of the pocket. This geometry enables a network of stabilizing interactions, including both hydrogen bonds and hydrophobic contacts.

Detailed modeling of the cryo‐EM density reveals that N14 forms a water‐mediated hydrogen bond with the backbone carbonyl oxygen of Pro646 (Figure 4; Video S1). The benzene ring of UTE‐156 extends into a narrow hydrophobic cavity, forming van der Waals interactions with the side chains of Leu482, Ile479, Ala655, and Ile656. Additional packing interactions involve the pyrazole ring carbons with the side chains of Leu526 and Arg662, and the pyrimidine ring with the side chains of Ala659 and Leu526.

The binding of UTE‐156 also induces local conformational changes in surrounding residues. Superimposition of our UTE‐156‐bound model with the ADP‐bound structure of VCP (PDB ID:5FTK) [18] revealed that the ATP binding pocket widens substantially upon UTE‐156 binding (Video S2). This rearrangement displaces key Walker A motif residues Gly521, Thr525, Leu526, Gly684, Ala685, Ile479, and Gly480, highlighting the conformational plasticity of the D2 active site. Importantly, UTE‐156 binding does not perturb the D1 nucleotide pocket, which remains capable of ADP binding (Figure 3B). This observation is consistent with prior work demonstrating that inhibition of the D2 ATPase domain, rather than D1, is sufficient to fully suppress unfoldase activity [35].

In addition to the hexameric assembly, cryo‐EM analysis also revealed a dodecameric VCP reconstruction at 2.4 Å resolution. The assembly comprises two hexamers in which their D2 rings are stacked back‐to‐back (Figure 3D). In the UTE‐156–bound dataset, the compound is clearly bound at the Cys522‐containing site within each protomer and adopts the same binding mode observed in the hexameric assembly (Figure 3E,F). Protomer conformations in the hexameric and dodecameric structures are highly similar, and in both oligomeric states, UTE‐156 binding is associated with the loss of nucleotide density in the D2 ATPase domain.

Analysis of the UTE‐156‐free (DMSO) control dataset collected under identical biochemical conditions revealed both hexameric (2.3 Å resolution) and dodecameric (2.1 Å resolution) VCP assemblies at comparable relative abundance (Figure S8). Notably, in the absence of UTE‐156, clear ADP density was present in both the D1 and D2 ATPase domains of the dodecameric assembly. Together, these observations indicate that dodecameric VCP assemblies exist under basal conditions and can accommodate nucleotide occupancy in D2. UTE‐156 binding is therefore compatible with both hexameric and dodecameric oligomeric states and correlates specifically with the displacement of nucleotide from the D2 pocket.

The binding mode of UTE‐156 shares some common characteristics with that of CB‐5083 (PDB ID: 7RLI) [36] (Figure S9). The benzylamino group of CB‐5083 engages the same hydrophobic cavity that accommodates the benzene ring of UTE‐156, forming a network of hydrophobic contacts with Leu482, Ile479, Leu526, Leu527, and Ile656. However, CB‐5083 relies on stabilizing hydrogen bonds to Thr688 and Asn660, whereas UTE‐156 lacks these interactions and instead achieves inhibition through covalent linkage to Cys522. These overlapping, yet distinct, binding modes illustrate how covalent engagement at Cys522 expands the chemical space for targeting the VCP D2 ATPase domain.

## 3 Discussion

VCP has long been recognized as a challenging but promising therapeutic target due to its central role in proteostasis. Efforts to inhibit VCP for cancer treatment have produced multiple inhibitors over the past decade, demonstrating that VCP is druggable but also highlighting significant hurdles in potency, selectivity, and pharmacological properties. Numerous classes of inhibitors have been described, including allosteric modulators such as NMS‐873 and UPCDC30245, competitive inhibitors like CB‐5083, and covalent inhibitors including NMS‐859, Eeyarestatin I, and PPA. Despite these efforts, the atomic basis for covalent inhibition of VCP has remained undefined.

Here we report the development and structural characterization of UTE‐156, a novel irreversible covalent small molecule that selectively modifies Cys522 within the D2 ATPase domain of VCP. Our biochemical and mass spectrometry data demonstrate that UTE‐156 engages this specific cysteine within VCP, and our cryo‐EM structures provide the first direct visualization of a covalent inhibitor occupying the D2 ATP‐binding pocket.

Although previous studies identified covalent compounds that can react with Cys522, such as PPA [30] and LC‐1028 [33], their mechanisms of binding and inhibition were inferred primarily from modeling, and no structural evidence of engagement was available. UTE‐156 builds on this work by providing structural validation that reveals how covalent occupancy at the D2 binding site blocks ATP binding and hydrolysis and how it remodels the pocket. Compared with the reversible inhibitor CB‐5083, UTE‐156 occupies an overlapping hydrophobic cavity but achieves inhibition through an entirely distinct covalent mechanism, demonstrating that both reversible and irreversible strategies can exploit the same structural region of VCP. These insights broaden the chemical space for targeting VCP and related AAA+ ATPases.

While UTE‐156 displays potent biochemical activity, its limited solubility, high metabolic clearance, and rapid glutathione reactivity restrict its suitability for in vivo applications. Importantly, the observed glutathione reactivity reflects the inherent electrophilicity of the alkynyl ketone warhead and does not contradict the site‐selective covalent modification observed within VCP, as demonstrated by peptide‐level MS/MS analysis. Although these properties may limit broader cellular use, they do not diminish the value of UTE‐156 as a mechanistic probe in biochemical and targeted cellular studies.

Importantly, these solubility and exposure challenges are not unique to UTE‐156 but are widely encountered across multiple classes of VCP inhibitors. In other reported cases, replacement of triazole or other heterocycles with alternative heteroaryl groups has improved solubility and in vitro ADME properties, often at the cost of reduced biochemical potency [13]. These observations highlight the inherent trade‐offs in VCP inhibitor optimization and emphasize the need for systematic structure–activity relationship (SAR) studies to balance potency with solubility, permeability, and metabolic stability.

Structure‐guided approaches have proven particularly valuable in this regard, as high‐resolution VCP structures reveal solvent‐exposed regions of the nucleotide‐binding pocket that can accommodate polar or solubilizing substituents without disrupting key target interactions. From a cellular perspective, improved solubility alone has not always translated into enhanced cellular activity, underscoring the importance of integrating solubility optimization with cellular uptake and intracellular target engagement. In this context, the VCP–UTE‐156 structure identifies solvent‐exposed regions of the D2 binding pocket that are distal to the covalent warhead and key hydrophobic interactions, suggesting opportunities to introduce solubilizing substituents or to replace metabolically labile moieties without compromising covalent engagement. Similar structure‐guided strategies have been successfully applied to competitive and allosteric VCP inhibitors, providing proof of concept that physicochemical properties can be improved through rational design [38].

The high‐resolution cryo‐EM structure of the VCP–UTE‐156 complex opens exciting opportunities for the rational optimization of the next generation of VCP inhibitors. Future efforts could focus, for example, on improving aqueous solubility and metabolic stability by modifying metabolically labile moieties or incorporating solubilizing groups, while preserving the key functional groups and covalent modification responsible for potency. The structural insights presented here establish a robust foundation for rational optimization of Cys522‐directed inhibitors with improved pharmacological profiles.

In addition to the hexameric assembly, a dodecameric VCP structure was resolved in which UTE‐156 is clearly bound at the Cys522‐containing site within each protomer, adopting the same binding mode observed in the hexameric structure. Importantly, cryo‐EM analysis of a UTE‐156‐free (DMSO) control dataset revealed a comparable population of dodecameric assemblies. In this control structure, ADP is present in both the D1 and D2 ATPase domains, suggesting that dodecamer formation reflects an intrinsic oligomeric property of recombinant VCP under these conditions.

Dodecameric VCP assemblies have been reported previously in a range of conditions, including those determined in the presence of CB‐5083 [23], NMS‐873 [30], the allosteric activator VAA1 [39], the disease‐associated R155H missense mutation [40], or by ADP depletion [41]. While some of these studies suggested that specific perturbations may favor higher‐order assembly, our observations indicate that dodecamerization can occur independently of such factors and may represent a stable equilibrium state.

The functional relevance of the dodecameric state remains unresolved. It may represent a storage or regulatory assembly, a structurally stable reservoir of VCP, or a conformation that influences cofactor recruitment and substrate accessibility in a context‐dependent manner. The repeated observation of dodecamers in both endogenous [42] and recombinant systems supports the possibility that this assembly may reflect a physiologically relevant state. Further work will be required to define the dynamics, regulation, and physiological role of VCP dodecamerization in vivo.

In summary, UTE‐156 represents a new class of high‐quality covalent chemical probe targeting the VCP/p97 AAA+ ATPase. Its high‐resolution structural characterization defines the first atomic model of covalent engagement at Cys522 and reveals how this modification inactivates the D2 motor and provides a powerful tool to study VCP's cellular functions and mechanisms. These findings advance our molecular understanding of VCP inhibition and open avenues for the development of next‐generation modulators of protein homeostasis.

## 4 Methods

### 4.1 Chemical Synthesis of UTE‐156

Step 1: Synthesis of tert‐butyl (3‐((6‐chloro‐2‐(methylsulfonyl)pyrimidin‐4‐yl)amino)phenyl)carbamate (3): to a stirred solution of 4,6‐dichloro‐2‐(methylsulfonyl)pyrimidine **1** (5 g, 22.02 mmol) in DMF (25 mL) were added 2,6‐lutidine (3.55 g, 33.0 mmol) and tert‐butyl (3‐aminophenyl)carbamate **2** (5.50 g, 26.4 mmol) at 0°C. The reaction mixture was slowly brought to room temperature and stirred for 30 min. After completion of the reaction (by TLC), the reaction mixture was diluted with cold water (100 mL) and extracted with ethyl acetate (3 × 50 mL). Combined organic layers were washed with brine (50 mL), dried over anhydrous Na_2_SO_4_, concentrated under reduced pressure. The crude product was purified by column chromatography [(silica‐gel,50‐60 mesh size), ethyl acetate in pet ether (50% to 60%) as an eluent] to obtain, tert‐butyl (3‐((6‐chloro‐2‐(methylsulfonyl)pyrimidin‐4‐yl)amino)phenyl)carbamate (6.1 g, 15.29 mmol, 70% yield) as an off white solid. ^1^H NMR (300 MHz, DMSO‐d_6_, δ): 10.49 (s, 1H), 9.50 (s, 1H), 7.80 (bs, 1H), 7.32–7.27 (m, 1H), 7.27 (t, J = 8.1 Hz, 1H), 7.14–7.12 (m, 1H), 6.96 (s, 1H), 3.81 (s, 3H), 1.48 (s, 9H); MS (ESI) 343.0 [(M‐t‐Bu)+H]. Step 2: Synthesis of tert‐butyl (3‐((6‐(1‐methyl‐1H‐pyrazol‐4‐yl)‐2‐(methylsulfonyl)pyrimidin‐4‐yl)amino)phenyl)carbamate (5): to a stirred solution of tert‐butyl (3‐((6‐chloro‐2‐(methylsulfonyl)pyrimidin‐4‐yl)amino)phenyl)carbamate (5 g, 12.54 mmol) in dioxane (10 mL) and water (2.2 mL) at room temperature, were added (1‐methyl‐1H‐pyrazol‐4‐yl)boronic acid (2.368 g, 18.80 mmol) and K_2_CO_3_ (5.20 g, 37.6 mmol) at room temperature. Nitrogen gas was purged through the reaction mixture at room temperature for 15 min. Pd(dppf).DCM (1.023 g, 1.254 mmol) was added to the reaction mixture and the reaction was heated at 60°C for 2 h. The reaction was monitored by TLC. On completion, the reaction mixture was filtered through celite bed and washed with EtOAc (50 mL × 3) then dried over anhydrous sodium sulphate, filtered, and concentrated under reduced pressure to afford the crude product. The crude product was purified by column chromatography (silica 100–200 mesh) using pet ether/ethyl acetate as eluting solvent to obtain, tert‐butyl (3‐((6‐(1‐methyl‐1H‐pyrazol‐4‐yl)‐2‐(methylsulfonyl)pyrimidin‐4‐yl)amino)phenyl)carbamate (3.5 g, 7.87 mmol, 63% yield) as a light yellow solid. ^1^H NMR (300 MHz, DMSO‐d_6_, δ): 10.17 (s, 1H), 9.47 (s, 1H), 8.42 (s, 1H), 8.04 (s, 1H), 7.78 (s, 1H), 7.36−7.33 (m, 1H) 7.24 (t, J = 8.1 Hz, 1H), 7.10−7.07 (m, 2H), 3.92 (s, 3H), 3.39 (s, 3H) 1.49 (s, 9H). MS (ESI) 445.2 (M+H). Step 3: Synthesis of N^1^‐(6‐(1‐methyl‐1H‐pyrazol‐4‐yl)‐2‐(methylsulfonyl)pyrimidin‐4‐yl)benzene‐1,3‐diamine Hydrochloride (6): to a stirred solution of tert‐butyl (3‐((6‐(1‐methyl‐1H‐pyrazol‐4‐yl)‐2‐(methylsulfonyl)pyrimidin‐4‐yl)amino)phenyl)carbamate (1.45 g, 3.26 mmol) in 1,4‐dioxane (10 mL) at 0°C was added HCl (4 m in dioxane; 0.496 mL, 16.31 mmol). The reaction mixture was allowed to warm to room temperature and stirred for 3 h. Reaction progress was monitored by TLC. Upon completion, the reaction mixture was concentrated under reduced pressure to afford the corresponding hydrochloride salt (compound 6), which was used directly in the next step without further purification as a white solid (1.20 g, 3.47 mmol, 88% yield).

MS (ESI):m/z 345.2 [M + H]^+^.Step 4:Synthesis of N‐(3‐((6‐(1‐methyl‐1H‐pyrazol‐4‐yl)‐2‐(methylsulfonyl)pyrimidin‐4‐yl)amino)phenyl)propiolamide UTE‐156 (8):to a stirred solution of N^1^‐(6‐(1‐methyl‐1H‐pyrazol‐4‐yl)‐2‐(methylsulfonyl)pyrimidin‐4‐yl)benzene‐1,3‐diamine hydrochloride 6 (70 mg, 0.183 mmol, 1.0 equiv) in DMF (1.0 mL) at 0°C were added pyridine (30 µL, 0.367 mmol, 2.0 equiv), propiolic acid (25 mg, 0.366 mmol, 2.0 equiv), and EDC·HCl (52 mg, 0.274 mmol, 1.5 equiv). The reaction mixture was allowed to warm to room temperature and stirred for 1 h. TLC analysis indicated formation of a new less‐polar product. The reaction mixture was diluted with EtOAc and extracted (2 × 10 mL). The combined organic layers were washed with brine, dried over anhydrous Na_2_SO_4_, filtered, and concentrated under reduced pressure. The crude product was purified by preparative TLC (EtOAc/hexanes, 1:1) to afford N‐(3‐((6‐(1‐methyl‐1H‐pyrazol‐4‐yl)‐2‐(methylsulfonyl)pyrimidin‐4‐yl)amino)phenyl)propiolamide (UTE‐156) as an amorphous white solid (35 mg, 48% yield). TLC:EtOAc/hexanes (7:3), Rf ≈ 0.30.^1^H NMR (400 MHz, DMSO‐d6, δ):10.84 (s, 1H), 10.23 (s, 1H), 8.42 (s, 1H), 8.03 (s, 1H), 7.91 (s, 1H), 7.52 (d, J = 7.2 Hz, 1H), 7.33 (t, J = 7.6 Hz, 1H), 7.23 (d, J = 7.6 Hz, 1H), 7.06 (s, 1H), 4.44 (s, 1H), 3.92 (s, 3H), 3.39 (s, 3H). ^1^ ^3^C NMR (125 MHz, DMSO‐d6, δ):165.2, 160.7, 157.3, 149.4, 138.8, 137.4, 138.3, 130.9, 129.0, 119.5, 116.0, 114.5, 111.4, 100.9, 78.0, 76.9, 39.3, 39.0 ppm. HRMS (ESI) *m/z*: [M + H]^+^ calcd for C_18_H_16_N_6_O_3_S, 397.432; found 397.107. Mp (DSC):136.3°C (endothermic peak).

### 4.2 WT VCP Expression and Purification

Full‐length wild‐type human VCP sequence with TEV cleavage site was sub‐cloned into pET‐151 vector with N‐terminal 6XHis tag. RIL *E. coli* competent cells were transformed with the plasmid and grown in 10 mL of Luria‐Bertani broth medium with ampicillin and chloramphenicol at 37°C overnight. The overnight growth cells were added to the ZY autoinducing media and grown at 37 °C for 3 h and then at 19 °C overnight. IPTG was then added to the media to induce the expression of the construct and after 3 h post‐induction the culture was harvested by centrifugation at 3,000 rpm for 15 min and the pellet was stored at ‐80°C. The pellet was thawed and resuspended in lysis buffer (25 mm Tris‐HCl pH 7.4, 450 mm NaCl, 20 mm imidazole, 1 mg/mL lysozyme, and a protease inhibitor cocktail:0.5 µg/mL leupeptin, 0.5 µg/mL aprotinin, 0.7 µg/mL pepstatin, and 16.7 µg/mL PMSF) for 40 min at 4°C, sonicated, and centrifuged at 20 000 rpm at 4°C for 45 min. The supernatant was bound to nickel‐NTA agarose (Qiagen) column followed by washing with Ni‐A buffer (50 mm Tris HCl pH 7.5, 500 mm NaCl, 50 mm Imidazole). The sample was eluted using Ni‐B buffer (50 mm Tris HCl pH 7.5, 500 mm NaCl, 300 mm Imidazole). The eluted protein was dialyzed overnight at 4°C in low sodium buffer (150 mm NaCl and 25 mm Tris‐HCl pH 7.5), followed by cleavage of the His tag by addition of TEV. Following cleavage of the His tag, the protein was purified by anion exchange chromatography (Hi Trap Q HP 5 mL, Cytiva) using a sodium gradient starting with the high sodium buffer (1 m NaCl and 25 mm Tris‐HCl pH 7.5) followed by a low sodium buffer (150 mm NaCl and 25 mm Tris‐HCl pH 7.5). The protein was further purified by size exclusion chromatography using a Superose 6 column with SEC buffer (100 mm NaCl and 25 mm HEPES buffer pH 7.4, 0.5 mm DTT, and 20 mm MgCl_2_), and the fractions were analyzed by SDS‐PAGE (Figure S10). Finally, the purified protein was concentrated, aliquoted, and snap frozen in liquid nitrogen, and stored at −80°C.

### 4.3 C522A VCP Expression and Purification

Full‐length C522A human VCP sequence with TEV cleavage site was sub‐cloned into pET‐151 vector with N‐terminal 6x‐His tag. The vector was transformed into *E. coli* Rosetta competent cells (50 µL), plated on LB agar supplemented with ampicillin (100 µg/mL) and chloramphenicol (34 µg/mL), and incubated overnight at 37°C. A single colony was used to inoculate a 2% starter culture, which was expanded into 5 L of Terrific Broth containing the same antibiotics. Cultures were grown at 37°C for 4 h until OD_600_ reached 0.76, and expression was induced with 0.5 mM IPTG at 20°C for 16 h. Cells were harvested by centrifugation at 6000 rpm for 10 min. Cells were lysed using a microfluidizer at 600 bar for three passes in lysis buffer (50 mm Tris‐HCl, 500 mm NaCl, 50 mm imidazole, 5 mm β‐mercaptoethanol, pH 7.5) supplemented with protease inhibitor cocktail (1:10 dilution). The lysate was clarified by centrifugation at 14 000 rpm for 40 min, and the supernatant was used for affinity purification. Ni‐affinity chromatography was performed using 15 mL of His60 Ni Superflow resin packed in an XK 16/20 column on an ÄKTA Go system. The column was regenerated with 0.5 m NaOH and equilibrated with Buffer A (50 mm Tris‐HCl, 500 mm NaCl, 2 mm TCEP, 50 mm imidazole, protease inhibitor cocktail, pH 7.5). The clarified lysate was loaded at 0.7 mL/min and washed sequentially with Buffer A, Buffer A + 5% Buffer B (25 mm imidazole), and Buffer A + 10% Buffer B (50 mm imidazole). Elution was performed using a linear gradient of 10% to 60% Buffer B (Buffer B: 50 mm Tris‐HCl, 500 mm NaCl, 2 mm TCEP, 50 mm imidazole, protease inhibitor cocktail, pH 7.5 + 0.5 m imidazole), followed by 100% Buffer B. Elution fractions were analyzed by SDS‐PAGE. The primary elution lot was buffer‐exchanged using 30 kDa Centricon filters and concentrated to 35 mL (8.8 mg/mL). The protein was subjected to TEV protease digestion at a 1:20 ratio (15 mg TEV), confirmed by mass spectrometry. After digestion, the sample was buffer exchanged to remove imidazole and applied to a Ni‐affinity column (HisTrap HP, 2 × 5 mL) on an ÄKTA Pure system. The flowthrough, containing the cleaved, tag‐free protein, was collected and analyzed. Final purification was achieved via size‐exclusion chromatography using a HiLoad 16/600 Superose 6 column equilibrated with buffer containing 25 mm HEPES, 100 mm NaCl, 2 mm TCEP, and 20 mm MgCl_2_ (pH 7.4). Two injections (∼5 mL each) were run at a flow rate of 0.5 mL/min, and fractions were analyzed by SDS‐PAGE.

### 4.4 ATPase Assay

The ATP assays for purified human VCP were performed using the ADP‐Glo assay kit from Promega Corporation (Cat # V9102) according to the manufacturer's instructions. UTE‐156, UTE‐330, CB‐5083, or LC‐1028 were diluted in DMSO with 1:3 serial dilutions for a total of ten different concentrations ranging from 0.2 µm to 10 mm in a compound dilution plate (384 well, Labcyte, Cat # LP‐0200). The kinase reaction was performed with 1× kinase reaction buffer (40 nm Tris base pH 7.5, 20 mm MgCl_2_), 0.1 mg/mL bovine serum albumin, distilled H_2_O, 40 nm recombinant VCP, and 20 µm ATP in a total assay volume of 20 µl. Briefly, 50 nL of different concentrations of UTE‐156, UTE‐330, CB‐5083, or LC‐1028 were added in assay plate (ProxiPlate‐384, Perkin Elmer, cat# 6008280) by ECHO (Echo Acoustic Liquid Handlers 650, Beckman Coulter) followed by the addition of 2.5 µl VCP and incubated for 60 mins at room temperature. After 60 min, 2.5 µl of ATP were added to all the wells (Max, Min, and the DRC wells). The plate was spun briefly for 1 min at 1000 rpm and further incubated at room temperature for 90 min. After incubation, 5 µl of ADP Glo reagent was added to the wells at room temperature. After 40 min, 10 µl of Kinase detection reagent was added per well and incubated for 60 min. Luminescence was measured using Tecan Spark with an integration time of 250 ms. Positive and negative controls were performed in 0.1% DMSO in the presence and absence of VCP kinases. Graphpad Prism software was used for data analysis.

### 4.5 Mass Spectrometry

Approximately 80 µg of purified human VCP protein was dissolved in 80 µl 1X PBS and incubated with UTE‐156 (10x final concentration) for 2 h. Then the reaction was quenched using formic acid. A UPLC‐MS method was developed for mass analysis of UTE‐156, using a 15‐min run at 0.3 mL/min with UV detection at 280 nm. Separation was achieved on a Waters Acquity Protein BEH C4 column (300 Å, 1.7 µm, 2.1 mm × 50 mm) at 70°C, with a mobile phase gradient from 95% to 5% aqueous (0.1% formic acid) over acetonitrile (0.1% formic acid). Analysis was performed on a Waters H‐Class Bio UPLC coupled to a Synapt G2‐Si MS in positive ESI mode (400–3000 m/z). Optimized MS parameters ensured efficient ionization and detection, supporting high‐sensitivity protein characterization.

Intact mass analysis for VCP + LC‐1028 was performed in the same way as described above and analyzed at 2 h (in 1X PBS buffer and ADP‐Glo buffer + 40 µm ATP) and 24 h (ADP‐Glo buffer + 40 µM ATP) after incubation.

### 4.6 PMF(MS/MS) for VCP's Cys Containing Peptides

50 µg of purified VCP protein was dissolved in 50 µl 1X PBS buffer and incubated with UTE‐156 (10x final concentration) for 2 and 24 h and the reaction quenched with formic acid. The sample was analyzed by LC‐MS (Water's QTOF) to determine its intact mass to confirm the formation of the covalent adduct. The VCP‐UTE156 adduct (∼45 µg in 45 µl 1X PBS), as well as a VCP only control (∼45 µg in 45 µl 1X PBS), were denatured with 25 µl of Rapigest SF (Waters) and incubated at 37°C for 45 min. The samples were then reduced with 10 mM DTT and incubated at 37°C for 45 min followed by alkylation with 15 mm Iodoacetamide at room temperature in the dark. Trypsin was used to digest the samples with a 1:20 enzyme: protein (w/w) ratio by incubating for 16 h at 37°C. Then the reactions were quenched with 0.1% formic acid. The obtained peptides were completely dried on a speed vac and desalted with C18 cartridge for analysis on high resolution mass spectrometry (Agilent 6545 QTOF) using LC‐MS/MS approach. Raw data obtained from LC‐MS/MS were searched against VCP's sequence using MassHunter BioConfirm Agilent software with adjustments in the following criteria: Mass tolerance (peptide level−30 ppm, fragment level−50 ppm), MS/MS confident level (at least 5 primary ions matched), Bio score > 30, and FDR < 1%. PMF(MS/MS) analysis for VCP + LC‐1028 was performed in the same way as described above.

### 4.7 CellTiter‐Glo Assay (CTG)

UTE‐156 was 2‐fold diluted in DMSO. 9 µL of diluted compound were transferred to an Echo plate. 120 nL of DMSO were transferred to maximum signal wells and 120 nL of respective concentrations of compound to minimum signal wells using ECHO and respective dose response curve concentrations in the rest of the plate as per the plate map. A549 cells and HCT116 cells were harvested from flasks and washed with PBS followed by the addition of 0.25% Trypsin. Cells were then centrifuged and resuspended in the respective media. The cells were counted and placed in a 384‐well black opaque bottom plate according to the plate map (1,300 cells/well for A549 and 600 cells/well for HCT‐116). The plate was incubated for 72 h at 37°C and then equilibrated to room temperature by placing on the bench for 20 min. After this time, 40 µL CTG were added. The plate was then shaken for 10 min on a plate shaker at 100 rpm. After a brief centrifugation, the plate was read on Tecan Spark and Envision with a sealer.

### 4.8 LogD

The lipophilicity (LogD) of test compounds was determined using the shake flask method at pH 7.4 with 100 mm phosphate buffer. The buffer was prepared by mixing 19 mL of 1 m KH_2_PO_4_ (prepared by dissolving 27.2 g in 200 mL water) with 81 mL of 1 m K_2_HPO_4_ (34.8 g in 200 mL water) and diluting the mixture to 1 L, with final pH adjusted to 7.4. Pre‐saturated phosphate buffer and octanol were prepared by combining equal volumes of octanol and phosphate buffer, shaking for up to 24 h, and allowing the mixture to settle undisturbed for 48 h to form two distinct layers. The upper octanol phase and the lower aqueous phase were separated and used as the saturated solvents. UTE‐156 and reference controls were prepared as 10 mm stock solutions in 100% DMSO. Internal standards were prepared by adding 10 µL of a 40 mg/mL tolbutamide stock and 25 µL of a 10 mg/mL telmisartan stock (both in DMSO) to 1 L of acetonitrile to achieve final concentrations of 400 ng/mL and 250 ng/mL, respectively. For the assay, 10 µL of 10 mm UTE‐156 stock was added to a 96‐deep‐well plate, followed by 495 µL of saturated octanol, and mixed for 5 min at 1000 rpm. Next, 495 µL of saturated phosphate buffer was added, and the plate was shaken at room temperature for 2 h at 1000 rpm on a thermomixer. After incubation, the plate was centrifuged at 4,000 rpm for 10 min to separate the layers. From each well, 200 µL of the octanol (upper) and buffer (lower) phases were carefully collected. For LC‐MS/MS analysis, 10 µL of the octanol layer was added to 30 µL of blank phosphate buffer, and 30 µL of the buffer sample was added to 10 µL of blank octanol to achieve matrix matching. Each mixture was vortexed for 5 min, then combined with 260 µL of acetonitrile containing internal standards. After centrifugation at 4,000 rpm for 10 min, 200 µL of supernatant was collected. Due to high response, both octanol and buffer fractions were diluted 5‐fold with internal standard–containing acetonitrile. Additional 10‐fold dilution was performed on control samples by mixing 20 µL of supernatant with 180 µL of acetonitrile. Samples were analyzed by LC‐MS/MS, and LogD values were calculated using the logarithm of the ratio of the octanol phase peak area (normalized to injection volume) to the buffer phase peak area (also normalized to injection volume).

### 4.9 Metabolic Stability in Cryopreserved Human and CD‐1 Mice Hepatocytes

Cryopreserved CD‐1 mouse or human hepatocytes were used to evaluate metabolic stability in vitro. In both cases, hepatocytes were thawed using a single‐step procedure adapted from BioreclamationIVT. Briefly, InVitroGRO HT medium was pre‐warmed to 37°C, and 49 mL were transferred to a sterile 50 mL conical tube. Vials of hepatocytes were thawed in a 37°C water bath and transferred into the pre‐warmed medium, followed by centrifugation at 50 × g for 5 min. The supernatant was carefully removed, and the cell pellet was resuspended in InVitroGRO KHB incubation medium. Cell viability was assessed using the trypan blue exclusion method, and viable cells were counted using a hemocytometer. UTE‐156 and assay controls were prepared as 10 mm master stock solutions in 100% DMSO. Intermediate working stocks (1 mm for test compounds and 3 mm for controls) were prepared by diluting the master stocks in DMSO, followed by final dilutions in incubation medium to yield 2 and 6 µm solutions, respectively. Internal standards were prepared in acetonitrile: tolbutamide (500 ng/mL) by diluting 12.5 µL of a 40 mg/mL DMSO stock into 1 L of acetonitrile, telmisartan (250 ng/mL) by diluting 25 µL of a 10 mg/mL stock, and quinidine (200 ng/mL) by adding 20 µL of a 10 mm stock to 1 L of acetonitrile. For the assay, 200 µL of final working stock (2 µm for UTE‐156 or 6 µm for controls) was mixed with 200 µL of hepatocyte suspension (2 × 10^6^ cells/mL) to give final concentrations of 1 µm (UTE‐156), 3 µm (control), and 1 × 10^6^ cells/mL. The mixtures were incubated at 37°C for 120 min with shaking at 250 rpm using a Heidolph Vibramax. At 0, 5, 10, 15, 30, 60, 90, and 120 min, 50 µL aliquots were withdrawn and quenched in 200 µL acetonitrile containing internal standards. Samples were vortexed and centrifuged at 4000 rpm for 10 min, and the resulting supernatants were analyzed by LC‐MS/MS for quantification of the compound.

### 4.10 Blood Plasma Partitioning

Blood‐to‐plasma partitioning of UTE‐156 was assessed using freshly collected whole blood from CD‐1 mice, anticoagulated with K_2_EDTA on the day of the experiment. UTE‐156 was prepared as 10 mm master stock solutions in 100% DMSO. Intermediate 1 mm stocks were generated by diluting 1 µL of the master stock in 9 µL of DMSO, and working stocks (100 µm) were prepared by diluting 2 µL of the 1 mm stock into 18 µL of 10% DMSO in Milli‐Q water. Internal standards were prepared by diluting 12.5 µL of a 40 mg/mL tolbutamide stock and 25 µL of a 10 mg/mL telmisartan stock (both in DMSO) into 1 L of acetonitrile to achieve final concentrations of 500 and 250 ng/mL, respectively. For the assay, 198 µL of whole blood and 99 µL of plasma were pre‐incubated separately at 37°C for 20 min. To each matrix, 2 µL (blood) or 1 µL (plasma) of the 100 µm test compound working solution was added, and samples were incubated at 37°C for 1 h with continuous shaking at 600 rpm on a thermomixer. At time 0 min, 25 µL aliquots of both plasma and blood were taken in duplicate and matrix‐matched by adding 25 µL of the opposite matrix. These were mixed with 50 µL of Milli‐Q water and precipitated with 300 µL of acetonitrile containing internal standards (tolbutamide 400 ng/mL and telmisartan 250 ng/mL). Samples were vortexed for 10 min at 1000 rpm, centrifuged at 4000 rpm for 20 min, and 100 µL of supernatant was diluted with 100 µL of water before LC‐MS/MS analysis. The same procedure was followed at the 60‐min time point. Additionally, at 60 min, blood samples were centrifuged to isolate plasma (T60 plasma), and 25 µL aliquots were again collected, matrix‐matched, and processed identically. LC‐MS/MS was used to quantify compound concentrations in each matrix, and the blood‐to‐plasma partition ratio was determined based on the concentration ratios in whole blood vs. plasma.

### 4.11 Kinetic Solubility

The kinetic solubility of UTE‐156 was determined in phosphate‐buffered saline (PBS, pH 7.4) using a shake‐and‐filter method. PBS buffer (10 mm, pH 7.4) was prepared by dissolving a commercially available PBS tablet in 190 mL of Milli‐Q water, adjusting the pH to 7.4 with 1N HCl or NaOH, and bringing the final volume to 200 mL. UTE‐156 and reference compounds were prepared as 10 mm DMSO stock solutions and stored at −80°C. Calibration curve standards ranging from 0.5 to 150 µm were prepared in 100% DMSO, beginning with a 150 µm solution made by diluting 6 µL of the 10 mm stock into 394 µL of DMSO, followed by serial dilutions. For the assay, 396 µL of PBS was pipetted into each well (in triplicate) of a 1.1 mL 96‐deep‐well plate, followed by 4 µL of 10 mm DMSO stock of UTE‐156 to achieve a final nominal concentration of 100 µm. Appropriate blanks (buffer + DMSO) and reference standards were included. The plate was sealed and incubated on an Eppendorf Mixmate shaker at 1000 rpm for 2 h at room temperature. After incubation, the plate was centrifuged at 4000 rpm (3220 × g) for 5 min, the supernatant was filtered through a 0.45 µm HTS filter plate, and the filtrate was collected into a fresh 96‐well plate for LC‐MS/MS analysis. Internal standards were prepared by adding 10 µL of a 40 mg/mL tolbutamide stock and 25 µL of a 10 mg/mL telmisartan stock (both in DMSO) to 1 L of acetonitrile to yield final concentrations of 400 ng/mL and 250 ng/mL, respectively. For analysis, 10 µL of the test filtrate was mixed with 10 µL of the opposite matrix (DMSO or PBS), and the mixture was added to 980 µL of acetonitrile containing internal standards. Samples were vortexed at 1000 rpm for 5 min, centrifuged at 4000 rpm for 10 min at room temperature, and 40 µL of the supernatant was diluted with 160 µL of acetonitrile containing internal standards before LC‐MS/MS analysis. Solubility values were calculated using matrix‐matched calibration curves. Dilution factors were adjusted as necessary based on signal response.

### 4.12 VCP‐UTE‐156 Cryo‐EM Sample Preparation and Data Collection

UTE‐156 was dissolved in 100% DMSO at room temperature to a concentration of 13.2 mm. Purified VCP was incubated with UTE‐156 for 5 h at 4°C at a final concentration of 7 µm VCP hexamer and 252 µM UTE‐156 (2% DMSO final concentration) in dilution buffer (25 mm of HEPES‐KOH pH 7.4, 100 mm of KOAc, 10 mm of MgCl_2_). UltrAuFoil R1.2/1.3 Au300 mesh (Quantifoil) EM grids were glow‐discharged two times for 30 s at 25 mA using a Pelco easiGlow unit (Ted Pella, Inc.). 3.5 µL of the incubated sample was applied to previously glow‐discharged grids and blotted for 2.5 s at 4°C and 91% humidity before being plunge frozen into liquid ethane using a Vitrobot Mark IV. Data collection of cryo‐EM movies was performed in a 300 kV Titan Krios (Thermo Fisher Scientific) equipped with a Gatan BioQuantum energy filter operated with a 20 eV slit width and a post‐GIF K3 direct electron detector (Gatan, Inc.), using SerialEM [43]. A total of 9,536 cryo‐EM movies were recorded at 105,000X nominal magnification. Each movie was recorded using counting mode for 2.5 s with a pixel size of 0.838 Å/pixel, 52.015 e/Å^2^ total electron dose, and 40 frames per movie. Defocus values ranged between −0.8 and −1.5 µm.

### 4.13 VCP‐UTE‐156 Cryo‐EM Image Processing

The collected 9536 movies were imported into CryoSPARC Live [44] and patch motion corrected and patch CTF estimated. The micrographs were exported into CryoSPARC [45] v4.6.2 and manually curated to discard the ones with poor CTF fit (5 Å cutoff). The accepted 7,852 micrographs were used to manually pick particles to create templates for template picking. A total of 822,393 particles were 4X binned extracted after inspection of the template picking job and taken to three rounds of 2D classification to discard junk particles. 405,256 particles were re‐extracted at box size of 400 pixels and subjected to another round of 2D classification. From this classification, two sets of particles were grouped: top views and side views. Each of the two groups were subjected to one round of 2D classification to separate top views and side views corresponding to hexamer and dodecamer. A total of 272,592 particles were obtained for the hexameric structure after regrouping and 117,690 for the dodecameric structure. Each group was independently taken to the Ab‐initio reconstruction. For the hexamer, the ab‐initio reconstruction was followed by non‐uniform refinement with C6 symmetry imposed with optimize per‐particle defocus and optimize per‐group CTF parameters enabled. Further heterogeneous refinement (C1 symmetry) was performed to discard junk particles. The obtained 204722 good particles were subjected to non‐uniform refinement (C6 symmetry) with optimize per‐particle defocus and optimize per‐group CTF parameters enabled, followed by referenced based motion correction, and finally another round of non‐uniform refinement (C6 symmetry) with optimize per‐particle defocus and optimize per‐group CTF parameters enabled, which resulted in a hexamer reconstruction at 2.8 Å resolution (Figures S11 and S12). The ab‐initio reconstruction for the dodecameric structure was followed by non‐uniform refinement with D6 symmetry imposed and with optimize per‐particle defocus and optimize per‐group CTF parameters enabled, followed by referenced‐based motion correction, and finally another round of non‐uniform refinement (D6 symmetry) with optimized per‐particle defocus and optimize per‐group CTF parameters enabled, which resulted in a dodecamer reconstruction at 2.4 Å resolution (Figures S11 and S12).

### 4.14 VCP‐UTE‐156 Model Building, Refinement, and Validation

The structure of a single protomer of VCP (PDB 5FTK) [18] with ADP at the D1 domain but without ADP at the D2 domain was rigid body fitted in the 2.8 Å hexamer map using ChimeraX [46, 47]. The structure was real‐space refined in Phenix v1.20 [48] with morphing and simulated‐annealing enabled. Detailed model building was performed using Coot v0.9.6 [49]. The obtained model was then rigid body fitted in the 2.4 Å dodecamer map using ChimeraX. The UTE‐156 ligand structure was built de novo and the geometry restraints were generated using eLBOW (as part of Phenix v1.20 [48]). This UTE‐156 was initially manually placed into the D2 nucleotide‐binding pocket based on the corresponding cryo‐EM density. The covalent bond between C522 and UTE‐156 was added in ChimeraX and the apply‐link.def file created. The covalently modified model (UTE‐156 included) was then subjected to real‐space refinement in Phenix with a supplement of UTE‐156 molecule restraint.cif file and the UTE156_VCP_link restraint.cif file (without simulated‐annealing or morphing enabled) using the dodecamer map. A detailed model was again manually refined in Coot v0.9.6 where also a C‐terminal extension for residues 764–774 was manually built de novo and real‐space refined using the dodecamer map and a water placed within the D2 binding pocket. These complexed protomer model was re‐checked against the hexamer map and used for the construction of both models (hexamer and dodecamer). The rest of the VCP symmetrically‐related subunits were added into the refined model using ChimeraX sym command (C6 for the hexamer model, D6 for the dodecamer model) and combined into single VCP(hexamer/dodecamer)‐UTE156‐ADP models. Both models were further validated using Molprobity [50, 51]. Model refinement statistics are summarized in Table ST1.

### 4.15 Spastin ATPase Assay

Purified WT human Spastin used in this assay was kindly provided by Chris Hill (University of Utah). ATPase activity was measured using the Malachite Green Phosphate Assay Kit (MilliporeSigma, Cat. No. MAK307‐1KT) according to the manufacturer's instructions. Purified WT human VCP was included as a positive control for AAA+ ATPase activity. UTE‐156 was dissolved in 100% DMSO at room temperature to a stock concentration of 4.078 mm. For each reaction, 58.15 µL of purified Spastin or VCP was dispensed into a 96‐well assay plate (Corning, Catalog number: 3370). Spastin and VCP reactions were prepared in 12 and 11 technical replicates, respectively. Proteins were incubated with 1.85 µL of UTE‐156 stock solution for 1 h at 4°C, resulting in final concentrations of 0.5 µm protein and 126 µm UTE‐156 in assay buffer (20 mm HEPES‐KOH, pH 7.4, 100 mm NaCl, 10 mm MgCl_2_). ATP hydrolysis was initiated by the addition of 20 µL of 1 mm ATP, followed by incubation for 15 min at 37°C. The reaction was terminated by the addition of 20 µL of malachite green working reagent and allowed to develop for 10 min at room temperature. Absorbance was measured at 620 nm using a BioTek Synergy Neo2 multimode microplate reader. Released inorganic phosphate from ATP hydrolysis was quantified using a phosphate standard curve generated in duplicate according to the manufacturer's instructions. Assay buffer alone served as a negative control. Data analysis was performed using Microsoft Excel. Statistical significance was determined using an unpaired two‐tailed t‐test with Welch's correction. ^*^ *p* < 0.05; ^**^ *p* < 0.01; ^***^ *p* < 0.001; ns, not significant.

### 4.16 VCP DMSO Control Sample Preparation and Cryo‐EM Data Collection

Purified VCP was mixed with DMSO in dilution buffer (25 mm of HEPES‐KOH pH 7.4, 100 mm of KOAc, 10 mm of MgCl_2_) at 4°C at a final concentration of 7 µm VCP hexamer and 2.4% DMSO. UltrAuFoil R1.2/1.3 Au300 mesh (Quantifoil) EM grids were glow‐discharged two times for 30 s at 25 mA using a Gatan Solarus Plasma Cleaner with oxygen and argon (O_2_/Ar) gases. 3.5 µL of sample were applied to previously glow‐discharged grids and blotted for 1.5 s at 4°C and 100% humidity before being plunge frozen into liquid ethane using a Vitrobot Mark IV. Data collection of cryo‐EM movies was performed at The New York Structural Biology Center (Simons Electron Microscopy Center) in a 300 kV Titan Krios (Thermo Fisher Scientific) equipped with a Selectris X energy filter operated with a 20 eV slit width with a Falcon IV direct electron detector operating in electron‐counting mode (Krios #7). A total of 4,487 movies were collected at 165,000X nominal magnification using Leginon [52]. Each movie was recorded using counting mode for 4.5 s with a pixel size of 0.7304 Å/pixel, 50.64 e/Å^2^ total electron dose, and 90 frames per movie. Defocus values ranged between −0.4 to −2.2 µm.

### 4.17 VCP DMSO Control Cryo‐EM Image Processing

The collected 4,487 movies were imported into CryoSPARC [45] v4.7.1 and patch motion corrected and patch CTF estimated. The micrographs were manually curated to discard the ones with poor CTF fit (5 Å cutoff). The accepted 3,967 micrographs were used to manually pick particles to create templates for template picking. A total of 616,647 particles were 4X binned extracted after inspection of the template picking job and taken to three rounds of 2D classification to discard junk particles. 299,516 particles were re‐extracted at a box size of 456 pixels. All particles were taken to Ab‐initio reconstruction with 2 classes. The 96,355 particles belonging to the dodecamer class were subjected to heterogeneous refinement with 2 classes to discard suboptimal particles. The remaining 79,406 particles were taken to non‐uniform refinement with D6 symmetry imposed and with optimize per‐particle defocus and optimize per‐group CTF parameters enabled. Then we performed referenced based motion correction, and finally another round of non‐uniform refinement (D6 symmetry) with optimize per‐particle defocus and optimize per‐group CTF parameters enabled, which resulted in a dodecamer reconstruction at 2.13 Å resolution. The 201,701 particles from the ab‐initio hexameric class were subjected to non‐uniform refinement with C6 symmetry imposed with optimize per‐particle defocus and optimize per‐group CTF parameters enabled. This was followed by referenced‐based motion correction, and finally another round of non‐uniform refinement (C6 symmetry) with optimize per‐particle defocus and optimize per‐group CTF parameters enabled. This resulted in a hexamer reconstruction at 2.27 Å resolution (Figures S13 and S14).

## Funding

This work was supported by National Institutes of Health grants R35 GM133772 awarded to P.S.S. and R01 CA293084 awarded to K.B.J. and P.S.S.

## Conflicts of Interest

The authors declare no conflicts of interest.

## Supporting information




**Supporting File**:advs74665‐sup‐0001‐Suppmat.docx

**Supporting File**:advs74665‐sup‐0002‐VideoS1.mp4

**Supporting File**:advs74665‐sup‐0003‐VideoS2.mp4

## Data Availability

Cryo‐EM density maps and corresponding models have been deposited to the Electron Microscopy Data Bank (EMDB) and Protein Data Bank (PDB), respectively. For VCP hexamer‐UTE‐156, PDB ID: 9YP6 and EMDB ID: EMD‐73285. For VCP dodecamer‐UTE‐156, PDB ID: 9YP8 and EMDB ID: EMD‐73287. For VCP hexamer (DMSO control) PDB ID: 10QR and EMDB ID: EMD‐75392; for VCP dodecamer (DMSO control) PDB ID: 10QQ and EMDB ID: EMD‐75391. This study does not include any original code. Additional information neccessary to reproduce or reanalyze the data presented in this work is available from the corrresponding author upon reasonable request.
