# Supplementary material for: Development and Structural Characterization of UTE‐156, a Covalent Inhibitor of the VCP/p97 AAA+ ATPase
Source: Adv Sci (Weinh). 2026 Mar 7;13(25):e20545. doi: 10.1002/advs.202520545 (PMC13137804; doi:10.1002/advs.202520545)
Supplement: Supplementary file 1 — Supporting File:advs74665‐sup‐0001‐Suppmat.docx [file ADVS-13-e20545-s003.docx]

Supporting Information

Development and structural characterization of UTE-156, a covalent inhibitor of the VCP/p97 AAA+ ATPase

Daniela Tamayo-Jaramillo, Subramanya Hegde, Xuan Jia, Kimberly Coffman, Hariprasad Vankayalapati, David Bearss, Kevin B. Jones, Alex W. Stark and Peter S. Shen*

**Table S1**. Cryo-EM data collection, refinement, and validation statistics.

| **Structure** | **VCP Hexamer – UTE-156** | **VCP Dodecamer – UTE-156** | **VCP Hexamer**  **– DMSO control** | **VCP Dodecamer – DMSO control** |
| --- | --- | --- | --- | --- |
| EM Databank Accession ID | EMD-73285 | EMD-73287 | EMD-75392 | EMD-75391 |
| Protein Data Bank Accession ID | 9YP6 | 9YP8 | 10QR | 10QQ |
|  | | |  |  |
| **Data Collection** | | |  |  |
| Microscope | Titan Krios | Titan Krios | Titan Krios | Titan Krios |
| Voltage (kV) | 300 | 300 | 300 | 300 |
| Detector | Gatan K3 | Gatan K3 | Falcon IV | Falcon IV |
| Data collection software | SerialEM | SerialEM | Leginon | Leginon |
| Magnification | 105,000X | 105,000X | 165,000X | 165,000X |
| Dose rate (e^-^/Å^2^/second) | 20.806 | 20.806 | 11.25 | 11.25 |
| Total frames | 40 | 40 | 90 | 90 |
| Total exposure (e^-^/Å^2^) | 52.015 | 52.015 | 50.64 | 50.64 |
| Defocus range (μm) | -0.8 to -1.5 | -0.8 to -1.5 | -0.4 to -2.2 | -0.4 to -2.2 |
| Pixel size (Å) | 0.838 | 0.838 | 0.7304 | 0.7304 |
|  | | |  |  |
| **Data processing** | | |  |  |
| Number of micrographs collected | 9,536 | 9,536 | 4,487 | 4,487 |
| Number of micrographs accepted | 7,852 | 7,852 | 3,967 | 3,967 |
| Symmetry imposed for refinement | C6 | D6 | C6 | D6 |
| Final number of particles | 204,722 | 117,690 | 201,432 | 79,312 |
| Map resolution FSC 0.143 (unmasked) (Å) | 3.2 | 2.9 | 2.8 | 2.6 |
| Map resolution FSC 0.143 (masked, corrected) (Å) | 2.8 | 2.4 | 2.3 | 2.1 |
|  | | |  |  |
| **Model refinement** | | |  |  |
| Initial model used (PDB code) | 5FTK | 5FTK | 5FTK | 5FTK |
| Map sharpening B factor (Å^2^) | 112.6 | 83.6 | 73.5 | 55.8 |
| Map correlation coefficient (CC mask) | 0.78 | 0.85 | 0.64 | 0.67 |
| *Model composition* | | |  |  |
| Non-hydrogen atoms | 34,842 | 69,684 | 34,758 | 69,804 |
| Protein residues | 4,404 | 8,808 | 4,392 | 8,820 |
| *Ligands* | | |  |  |
| ADP | 6 | 12 | 12 | 24 |
| UTE | 6 | 12 | 0 | 0 |
| *R.m.s deviations* | | |  |  |
| Bond lengths (Å) | 0.005 (12) | 0.005 (24) | 0.006 (24) | 0.006 (48) |
| Bond angles (°) | 1.201 (6) | 1.201 (12) | 1.134 (6) | 1.129 (12) |
| *Validation* | | |  |  |
| MolProbity score | 2.13 | 2.15 | 2.18 | 2.13 |
| Clashscore | 16.84 | 17.31 | 16.81 | 15.30 |
| Poor rotamers (%) | 0.48 | 0.48 | 0.11 | 0.09 |
| *Ramachandran plot* | | |  |  |
| Favored (%) | 93.93 | 93.88 | 92.95 | 93.19 |
| Allowed (%) | 6.07 | 6.12 | 7.05 | 6.81 |
| Disallowed (%) | 0.00 | 0.00 | 0.00 | 0.00 |
| C-beta deviations (0.25 Å) | 0.00 | 0.00 | 0.00 | 0.00 |
| CaBLAM outliers (%) | 3.99 | 3.99 | 3.87 | 3.58 |
| EMRinger score | 1.87 | 2.86 | 2.47 | 2.94 |

| Sequence | Site of modification | Sequence  (start-end) | Observed m/z | FDR (%) | Modification |
| --- | --- | --- | --- | --- | --- |
| EAVCIVLSDDTCSDEKIR | C77 | 66-83 | 703.9976 | 0 |  |
| EAVCIVLSDDTCSDEKIR | C77 + LC-1028 | 66-83 | 724.349 | 7.28 | 0.42 % |
| LGDVISIQPCPDVK | C105 | 96-109 | 770.9068 | 0 |  |
| LGDVISIQPCPDVK | C105 + LC-1028 | 96-109 | 645.0074 | 0 | 0.05 % |
| VVETDPSPYCIVAPDTVIHCEGEPIKR | C174 | 165-191 | 771.1281 | 0 |  |
| EDEEESLNEVGYDDIGGCRK | C209 | 192-211 | 771.9953 | 0 |  |
| LADDVDLEQVANETHGHVGADLAALCSEAALQAIR | C415 | 390-424 | 918.9543 | 0 |  |
| GVLFYGPPGCGK | C522 | 513-524 | 626.3134 | 0 |  |
| GVLFYGPPGCGK | C522 + LC-1028 | 513-524 | 548.6117 | 0 | 0.37 % |
| AIANECQANFISIK | C535 | 530-543 | 789.9013 | 0 |  |
| AIANECQANFISIK | C535 + LC-1028 | 530-543 | 657.6677 | 0 | 0.04 % |
| QAAPCVLFFDELDSIAK | C572 | 568-584 | 962.4805 | 0 |  |
| QAAPCVLFFDELDSIAK | C572 + LC-1028 | 568-584 | 772.7194 | 0 | 0.04 % |
| MTNGFSGADLTEICQR | C691 | 678-693 | 900.407 | 0 |  |
| MTNGFSGADLTEICQR | C691 + LC-1028 | 678-693 | 851.0691 | 7.53 | 0.18 % |

**Table S2. PMS MS/MS analysis for VCP + LC-1028.** PMS MS/MS analysis showing the mass of cysteine containing peptides with and without covalent modification. Yellow rows indicate detected peptides modified by LC-1028. Only 0.5% modified cysteines were detected suggesting a reversible mechanism of inhibition for LC-1028.

**Figure S1. Characterization of UTE-156 by ^1^H and ^13^C NMR spectroscopy**. (A) ^1^H NMR spectrum of compound 8 (UTE-156) recorded at 400 MHz, DMSO-d6; chemical shifts are reported in parts per million (ppm) relative to residual solvent. (B) ^13^C NMR spectrum of compound 8 (UTE-156) recorded at 125 MHz, DMSO-d6; chemical shifts are reported in parts per million (ppm).


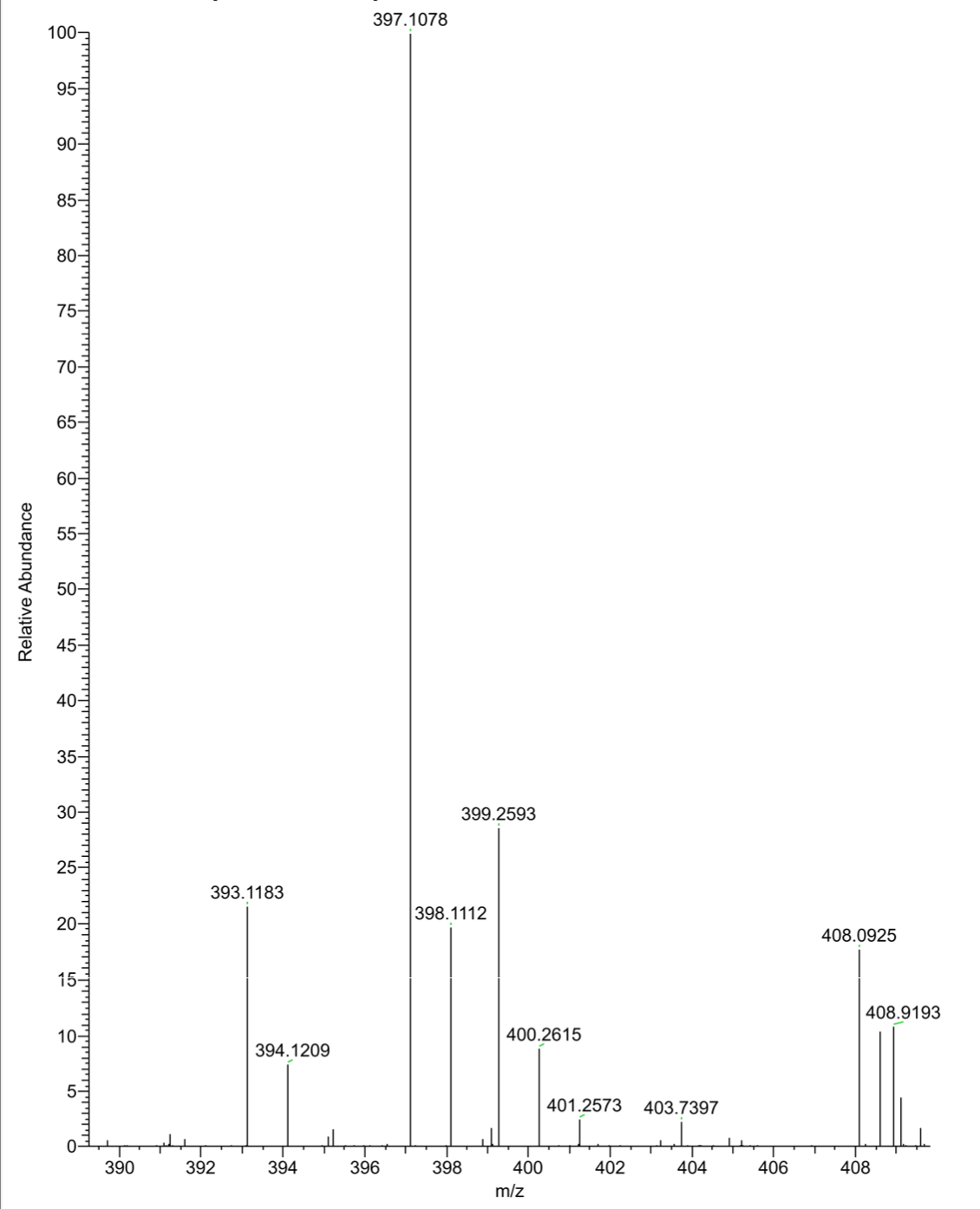


**Figure S2.** Characterization of UTE-156 by high-resolution mass spectrometry showing the molecular ion peak.


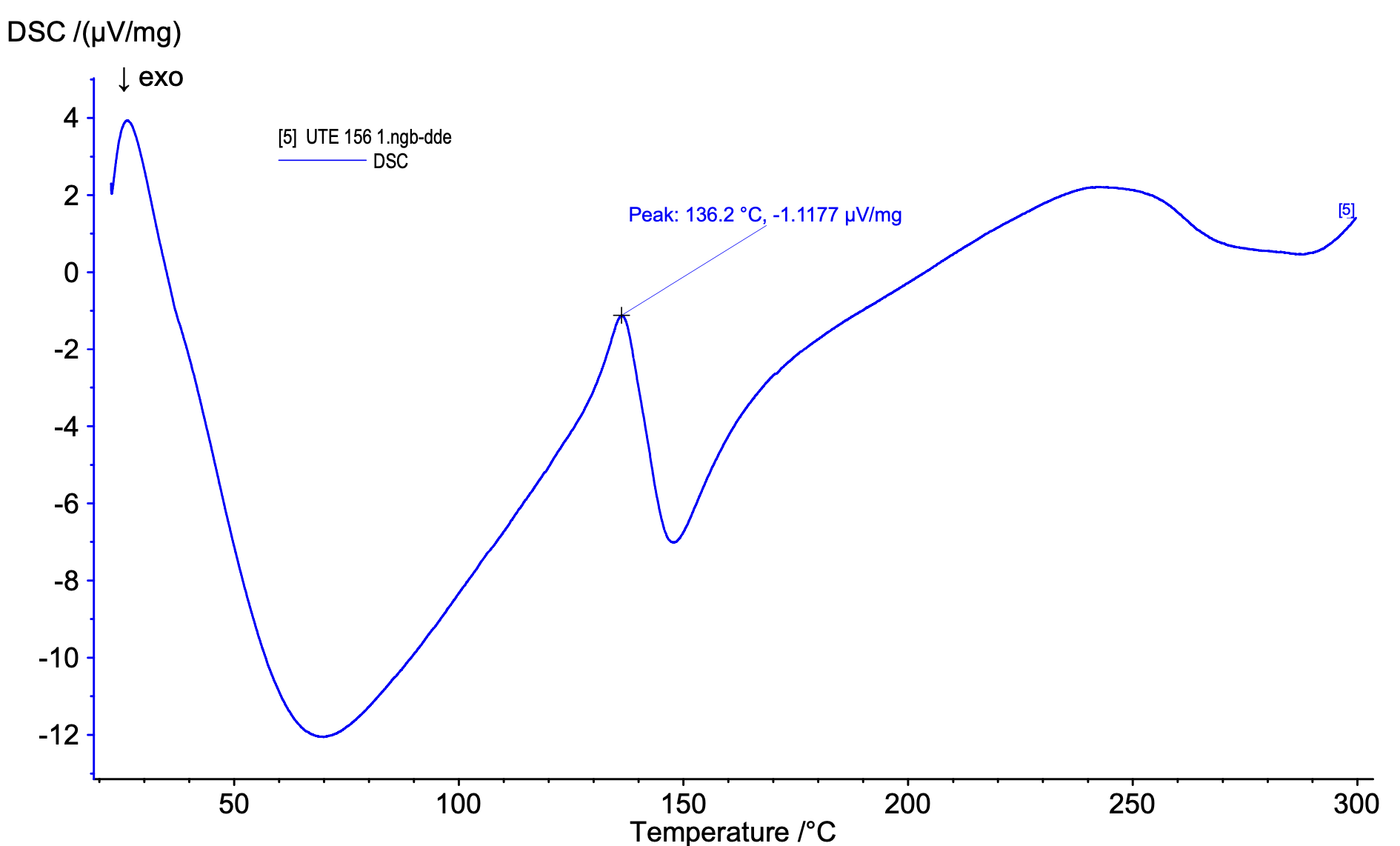


**Figure S3.** Differential scanning calorimetry (DSC) thermogram of compound 8 (UTE-156)

**Figure S4.** Intact mass spectrometry confirms selective modification of VCP at Cys522 by UTE-156. (A) Intact mass of purified human WT VCP (90379.1 Da), (B) After incubation of purified human WT VCP with UTE-156 we observed a single adduct (90786.0 Da) corresponding to mass shift of +395 Da, consistent with the mass of UTE-156, thus indicating complete modification of the protein by one molecule of the compound.

**Figure S5**. **The parent scaffold (UTE-330) lacking the alkyne warhead exhibited minimal inhibition of VCP’s ATPase activity**. The observed potency of UTE-156 is largely attributable to its electrophilic warhead, as demonstrated by UTE-330 ATPase activity analysis (A) Chemical structure of UTE-330. (B) Dose titration in the ADP-glo ATPase assay for VCP using compound UTE-330. UTE-330 shows and IC_50_>10^3^. UTE-156 ADP-glo ATPase assay results are displayed for comparison.

**Figure S6. LC-1028 showed activity consistent with predominantly reversible behavior**. Intact mass of purified human WT VCP (90379.1 Da) + LC-1028 after 2 h (A) or 24 h (B) incubation in ADP-Glo Buffer + 40 μM ATP. No adduct corresponding to mass shift consistent with the mass of LC-1028 was observed, thus indicating a predominantly reversible behavior. C) Intact mass of purified human WT VCP (90379.1 Da) + LC-1028 after 2 h incubation in 1X PBS buffer, pH 7.4.

**Figure S7. UTE-156 binding does not alter the ATPase activity of other AAA+ family member Spastin.** (A) ATPase activity of purified WT human Spastin in the absence and presence of UTE-156, p = 0.0517. (B) ATPase activity of purified WT human VCP under the same conditions p = 0.00194. *p < 0.05; **p < 0.01; ***p < 0.001; ns, not significant.

**Figure S8.** **Structural characterization of VCP-DMSO control** A) Model of VCP hexamer (DMSO control) displayed from side view showing D1 and D2 motor domains and respective binding sites for ADP. B) Close view of the D1 binding pocket with refined model for VCP (purple) and ADP (yellow) with cryo-EM reconstruction density of VCP hexamer DMSO control (gray mesh) C) Close view of the D2 binding pocket with refined model for VCP (blue) and ADP (yellow) with cryo-EM reconstruction density of VCP hexamer DMSO control (gray mesh). D) Model of VCP dodecamer (DMSO control) displayed from side view showing D1 and D2 motor domains and respective binding sites for ADP. B) Close view of the D1 binding pocket with refined model for VCP (purple) and ADP (yellow) with cryo-EM reconstruction density of VCP dodecamer DMSO control (gray mesh) C) Close view of the D2 binding pocket with refined model for VCP (blue) and ADP (yellow) with cryo-EM reconstruction density of VCP dodecamer DMSO control (gray mesh). All maps shown in this figure are displayed at a contour level of 2.5 σ.

**Figure S9. Structural comparison of UTE-156 and CB-5083 binding to VCP**. A) Model of CB-5083 binding to VCP nucleotide-binding pocket D2 (PDB ID: 7RLI). B) Refined model of VCP (blue) and UTE-156 (green).

**Figure S10.** **Size-exclusion chromatography (SEC) and SDS-PAGE of WT VCP purification**. (A) SEC elution profile showing the separation of WT VCP using a Superose 6 column. (B) Coomassie Brilliant Blue Stained SDS-PAGE gel of the fractions from the elution peak at 13.86 ml. The main band at 89 KDa corresponds to VCP protomer under the denaturing gel.

**Figure S11. Image processing workflow of VCP-UTE-156 particles.** (A) Representative cryo-EM micrograph, (B) Representative reference-free 2D classes, and (C) processing workflow for hexamer and dodecamer particles.

** Figure S12.** **Cryo-EM validation of VCP-UTE-156 dodecamer (A) and hexamer (B) reconstructions**. Upper left, local resolution heat maps; lower left, gold-standard FSC plots; upper right, particle orientation distribution; lower middle, particle orientation assignments; lower right, detailed view of the D2 region (residues 513–535), encompassing Cys522, fitted into the cryo-EM density map. Contour level 6 σ.

**Figure S13. Image processing workflow of VCP-DMSO control.** (A) Representative cryo-EM micrograph, (B) Representative reference-free 2D classes, and (C) processing workflow for hexamer and dodecamer particles.

**Figure S14.** **Cryo-EM validation of VCP-DMSO control dodecamer (A) and hexamer (B) reconstructions**. Upper left, local resolution heat maps; lower left, gold-standard FSC plots; upper right, particle orientation distribution; lower right, detailed view of the D2 region (residues 617-625) fitted into the cryo-EM density map. Contour level 8 σ.

**VIDEO LEGENDS**

**Video S1.** 360° rotational view of UTE-156 bound at the D2 binding pocket. Residues involved in non-covalent interactions with UTE-156 are highlighted in pink, while water-mediated hydrogen bonds are shown in green.

**Video S2.** Conformational rearrangement of the VCP D2 nucleotide binding pocket upon UTE-156 binding. The video shows a morph between the ADP bound state (PDB ID: 5FTK) and the UTE-156 bound state (blue), highlighting residue movements across the D2 pocket. The overall RMSD for the D2 pocket is 2.2 Å, with some deviations as large as 5.2 Å.
